# Use of the Biphasic ^13^C-Sucrose/Glucose Breath Test to Assess Sucrose Maldigestion in Adults with Functional Bowel Disorders

**DOI:** 10.1155/2016/7952891

**Published:** 2016-08-08

**Authors:** Antone R. Opekun, Albert M. Balesh, Harold T. Shelby

**Affiliations:** ^1^Division of Gastroenterology and Hepatology, Department of Internal Medicine, Baylor College of Medicine, Houston, TX 77030, USA; ^2^Division of Gastroenterology, Nutrition and Hepatology, Department of Pediatrics, Baylor College of Medicine, Houston, TX 77030, USA

## Abstract

Sucrase insufficiency has been observed in children with of functional bowel disorders (FBD) and symptoms of dietary carbohydrate intolerance may be indistinguishable from those of FBD. A two-phase ^13^C-sucrose/^13^C-glucose breath test (^13^C-S/GBT) was used to assess sucrase activity because disaccharidase assays are seldom performed in adults. When ^13^C-sucrose is hydrolyzed to liberate monosaccharides, oxidation to ^13^CO_2_ is a proportional indicator of sucrase activity. Subsequently, ^13^C-glucose oxidation rate was determined after a secondary substrate ingestion (superdose) to adjust for individual habitus effects (Phase II). ^13^CO_2_ enrichment recovery ratio from ^13^C-sucrose and secondary ^13^C-glucose loads reflect the individualized sucrase activity [*Coefficient of Glucose Oxidation for Sucrose *(CGO-S)]. To determine if sucrase insufficiency could be a factor in FBD, ^13^C-S/GBT was validated using subjects with known sucrase gene mutation status by comparing ^13^CO_2_-breath enrichment with plasma ^13^C-glucose enrichment. ^13^C-S/GBT was used to assess sucrose digestion in FBD patients and asymptomatic controls. ^13^CO_2_-breath enrichment correlated with the appearance of ^13^C-sucrose-derived glucose in plasma (*r* ^2^ = 0.80). Mean, control group CGO-S-enrichment outcomes were 1.01 at 60′, 0.92 at 75′, and 0.96 at mean 60′–75′ with normal CGO-S defined as >0.85 (95% C.I.). In contrast, FBD patients demonstrated lower CGO-S values of 0.77 at 60′, 0.77 at 75′, and 0.76 at mean 60′–75′ (*Chi Square: 6.55*; *p* < 0.01), which points to sucrose maldigestion as a cause of FBD.

## 1. Background

Functional bowel disorders (FBD) have an estimated prevalence of 10–21% of the general population for which the pathophysiology remains elusive [1]. As outlined by Rome III criteria, functional conditions include symptoms of recurrent abdominal discomfort (pain and bloating) associated with changes in bowel habits (diarrhea, constipation, or both) that persist for long periods in the absence of organic pathology [2, 3]. There is considerable overlap in the manner in which patients present with FBD and irritable bowel syndrome; often features of bloating and abdominal distension predominate without fully meeting specific criteria [4, 5]. The symptom complex appears similar to many conditions, including celiac disease, motility disorders, infectious enteritis, food allergies, inflammatory bowel disease, microscopic colitis, small bowel bacterial overgrowth syndrome, lactose intolerance, and nutrient malabsorption [6]. Some individuals seem to be disproportionally hypersensitive, and, at rare times, the discomfort may be incapacitating, having been equated to symptoms produced by someone with a bowel obstruction [7]. The diagnosis is based on exclusion of other organic disease entities [8], and without specific biomarkers, differentiating organic disease can be challenging [9, 10]. Many patients with functional symptoms report having experienced symptoms since childhood, which implies that a genetic predisposition may exist [11], but the risk-genes have not yet been validated [12]. The onset of first symptoms has often been associated with prior gastroenteritis and stressful life events [13], but the specific triggers, molecular mechanisms, and neurogut targets remain obscure such that the treatment approach largely aims to relieve symptoms in the absence of a specific etiology. The role of dietary factors in FBD appears important and understudied [7].

Up to 65% of FBD patients report symptoms exacerbated by meals with or without specific dietary intolerances [15, 16]. However, it is not clear what role food allergies may play in FBD, but it appears to be minimal [17]. A significant portion of pediatric patients evaluated for unexplained chronic abdominal pain were found to have decreased mucosal disaccharidase activity [18]. Patients with specific carbohydrate intolerance, such as lactose, frequently report symptom improvement with dietary restriction, and the reintroduction of those substrates (a so-called* dietary challenge*) results in symptom exacerbation, serving to confirm clinical suspicions [19, 20]. Since carbohydrates collectively provide approximately 60–70 percent of the daily dietary caloric load, one could postulate that even some degree of maldigestion could be a significant putative FBD factor [21].

Normal sucrose digestion to glucose and fructose and transporter-mediated monomer absorption that follows have been well-described [22–24]. The sequence of events that occurs in lactose intolerance is a paradigm for other malabsorptive circumstances, including starches and sucrose. Other oligosaccharides also require luminal, mucosal enzyme hydrolysis by additional gene products, and substrates include maltose, maltotriose, and maltodextrin with *α*-1,4- and *α*-1,6-glycosidic linkages [25]. Sucrase and isomaltase, chromosome 3 gene product, handle the bulk of sucrose and dextrin digestion (an incomplete, solubilized postpancreatic alpha-amylase hydrolysate) with a contribution by maltase-glucoamylase, chromosome 7-related gene product [26]. When undigested/malabsorbed nutrients pass distally to the colon where microbiota fermentation occurs, gas and short-chain fatty acids are produced which, in turn, diminish water reabsorption, induce inflammation, alter colonic motility, and theoretically bring on abdominal discomfort [27]. It is not clear if the observed gut dysbiosis is a consequence of maldigestion or is causally related to the symptoms.

The idea that disaccharide maldigestion, such as sucrose, could be causally involved in FBD is difficult to discount due to the striking similarity of symptoms [28, 29] and requires innovative approaches. The use of the breath-hydrogen test to evaluate maldigestion has only been helpful in some cases due to nonspecificity and poor sensitivity [30–32]. Breath-hydrogen production is dependent upon colonic microbiota and it is difficult to predict if the floras, in any patient, are hydrogen producers. Alternatively, when a link between carbohydrate maldigestion and GI symptomatology is suspected, specific approaches could include the use of endoscopic biopsy direct assays for sucrase, maltase, and palatinase (a surrogate for isomaltase). However, mucosal enzyme assays are onerous to perform, may not represent the confluent digestive capacity of the small intestine, and are often considered too expensive.

This study investigated the possibility of intestinal sucrase insufficiency as a contributing factor in FBD (functional bloating type) by using stable (*nonradioactive*) isotope tracer-labeled sucrose breath testing. Specifically, this study asked if sucrase insufficiency, as measured by an innovative two-phase ^13^C-sucrose/^13^C-glucose breath testing could, in part, explain the symptoms of FBD.

## 2. Methods

### 2.1. Study Design

This study was a prospective, open-label survey of adult patients with FBD as defined by Rome criteria and control subjects who presented to Baylor Clinic during a three-month period and subsequently underwent testing using biphasic ^13^C-S/GBT. A validation substudy was also performed to demonstrate that the biphasic breath test measures sucrose digestion. Additionally, the substudy evaluated four nondiabetic adult women with known sucrase-isomaltase genotypes to validate biphasic ^13^C-S/GBT.

### 2.2. Ethics

This study was performed in accordance with the Declaration of Helsinki and approved by the Institutional Review Board at Baylor College of Medicine under protocol H-31197. The Institutional Review Board at Baylor College of Medicine is organized, operates, and is registered with the United States Office for Human Research Protections according to the regulations codified in the United States Code of Federal Regulations at 45 CFR 46 and 21 CFR 56. The BCM IRB operates under the BCM Federal Wide Assurance number 00000286. Written documentation of informed consent was obtained from all participants and, in addition to consent for prospective study participation, explicitly permits the use of health information obtained from health records including diagnoses, medications, laboratory data (exclusive of the whole genotype), and demographic information including age, gender, race, and ethnicity; such authorization does not have an expiration date.

### 2.3. Study Population

Twenty-two subjects participated in this pilot study. Subjects were either asymptomatic healthy controls or symptomatic test subjects that met inclusion criteria for clinical diagnosis of FBD-functional bloating by Rome III criteria (FBD group: *n* = 11; 8 women and 3 men, 24–66 years of age) and in otherwise good health (Table 1).

Affected patients reporting any alarming issues, such as gastrointestinal bleeding, were not referred for study participation. Other exclusion criteria included, pregnancy, current lactation, allergy to food coloring, other causes of abdominal pain or altered bowel habits (e.g., celiac disease, pancreatitis, malignancy, or inflammatory bowel disease), history of diabetes mellitus, gastroparesis, recent febrile illness (5 days prior to the study), ingestion of an investigational drug or placement of an investigational device 30 days prior to the study start date, and absence of the mental capacity necessary to comprehend the protocol. Symptom severity to assess FBD was determined by patient reports, behavior of symptoms, and impact on health-related quality of life [14]. Symptom severity was scored according to the following summary definitions:* mild*: aware of signs or symptoms but maintained a good health-related quality of life;* moderate*: interfered with activities of daily living; or* severe*: marked impairment with psychological comorbidities and requires specialty medical intervention. There was no interventional component to this pilot study, and no long-term follow-up was done.

The asymptomatic control group (*n* = 11; eight women and three men, 22.8–60.8 years of age) was recruited from the student body and institutional staff at Baylor College of Medicine, none of whom met any criteria for FBD; all had a long history of tolerating sucrose in generous portions without inducing symptoms. The control subjects had no prior history of functional GI disorder, congenital sucrase-isomaltase deficiency, or irritable bowel syndrome. All reported an absence of current GI signs and symptoms, and all were in a state of excellent health.

To validate the breath test, four additional nondiabetic adult women were recruited as subjects for a substudy based upon their sucrase-isomaltase (SI) genotype (mutation status) and detailed self-reported dietary sucrose tolerance. As described below, ^13^CO_2_ breath test enrichment was compared with ^13^C-plasma enrichment since the only source of the tracer could be sucrose digestion with resultant monosaccharide absorption. One subject was symptomatic and homozygous for congenital sucrase-isomaltase (SI) deficiency, two subjects were heterozygous for congenital sucrase-isomaltase deficiency in the sucrase gene domain and moderately symptomatic, and one subject was sucrose tolerant (completely asymptomatic) without any of the known mutations in the sucrase-isomaltase gene (3q25.2-q26.2).

### 2.4. Description of Study and Breath Testing

Commercial Mylar® breath collection bags (1.3 L and 0.25 L) fitted with one-way valves (*Otsuka Electronics, Tokyo, Japan*) were used. Mouthpieces were fashioned from drinking straws to facilitate inflation by study participants and establish airtight connections between airway and collection bags to reduce the possibility of room air cross-contamination. Each kit contained five bags labeled for timed collections: one large bag for baseline collection for repeated reference enrichment comparisons and four test sample bags for 60-minute and 75-minute breath collections postsucrose and postglucose substrate ingestions (*delta-over-baseline*). Measured oxidation values typically peak between these time points in normal subjects at oral tracer dosage levels.

^13^C-tracer isotopes (*Isotec, Div. of Sigma-Aldrich*, Miamisburg, OH) and aqueous excipients were prepared and labeled under a clean Class 5 bench hood in 100 mL volumes [*MediPak USP Sterile Water* (37-6250),* McKesson Medical-Surgical*, Richmond, VA]. Dosed tracers and aqueous excipients composing the substrates were weighed analytically and mixed by combining 25.0 mg uniformly labeled ^13^C-sucrose and 125.0 mg uniformly labeled ^13^C-glucose with 15.0 grams unlabeled sucrose and 15 grams unlabeled glucose, respectively, as challenge loads. The unlabeled challenge-load gram-weight was selected because it approximates a threshold unlikely to induce symptoms or delay gastric emptying in most adults. To avoid confusion, substrates were color-coded using McCormick® food coloring (blue for sucrose and pink for glucose) to match the color-coordinated sample collection bags and flavored, respectively, with Adams® strawberry and coconut extracts for taste and aroma. All substrates were frozen at −20°C until needed in Nalgen® polyethylene bottles. Breath testing was performed in the clinic, home, or laboratory setting in the early morning hours before 12 p.m. after an overnight fast.

At study initiation, subjects were asked to hold their breath for a 10-second count and then inflate the reference breath sample for isotopic comparison later with timed breath samples. ^13^C-sucrose substrate solution with 15% excipient was then ingested (at time 0). At 60 minutes post-^13^C-sucrose ingestion, a breath sample was collected, followed by another breath sample at 75 minutes. Immediately after* Part I* sample collections related to sucrose ingestion were complete, a comparative ^13^C-glucose substrate solution was ingested (*Part II of the biphasic breath test*) to assess the inherent rate of metabolism for free glucose. This was used for comparison with* Part I* outcomes (see Section 2.5). The superdose (125 mg, 5 times the ^13^C-sucrose dose) was intended to dilute (mask) any residual ^13^CO_2_ carryover effects from the sucrose and to assess inherent body oxidation capacity. As before,* Part II* breath samples were collected at 60 minutes and 75 minutes after ^13^C–glucose substrate ingestion and results were adjusted by a factor of 0.2x to account for superdosing and to compare, pairwise, ^13^C–sucrose oxidation data with ^13^C–glucose data. Postprocedure breath test kits were promptly returned to the laboratory for analysis.

Measurement of ^13^CO_2_ sample enrichment was performed for each 60-minute and 75-minute breath collection using ^13^CO_2_ mass dispersive infrared spectrometer (*POCone®, Otsuka Electronics*, Tokyo, Japan). Each timed sample was analytically compared with the baseline sample (common, presubstrate ingestion sample) and breath test data were expressed as a ratio of the change in ^13^CO_2_ enrichment (*delta*‰) between the baseline sample and the periodically timed, postsubstrate ingested samples [delta‰ over baseline (DOB) at 60 and 75 minutes (DOB 60′) and (DOB 75′)].

### 2.5. Calculations

Because of body habitus and age-related variations in carbon dioxide production [33], ^13^CO_2_ oxidation value for ^13^C-glucose (Part II) was used to adjust for different intersubject ^13^C-sucrose (Part I) oxidation rates. Oxidized components derived from ^13^C-sucrose (fructose and glucose) were compared to oxidized enrichment values and expressed components derived from the secondary ^13^C-glucose superdose (1/5  × glucose enrichment). After mathematical adjustment for the superdosing of glucose, the enrichment ratio of ^13^CO_2_ was attributed to sucrose digestion, absorption, and metabolism. (*Note: glucose requires no further digestion.*) All glucose oxidation-related values were universally adjusted by a standard factor (1.12x) to account for minor inherent increased rate of fructose metabolism as compared with glucose (no isomerization needed) and the 17% proportionally greater atomic weight of sucrose. The ratio of two ^13^CO_2_ enrichment values was stated as the Coefficient of Glucose for Sucrose (CGO-S).

### 2.6. Validation

Considering the tremendous resources that would be required to conventionally validate the ^13^C-sucrose breath test using duodenal/jejunal biopsies obtained from adult patients coincidentally undergoing gastroduodenal endoscopy, an alternative validation substudy approach was undertaken in four genetically distinct subjects. The subjects were identified based upon their sucrase-isomaltase (SI) genotype and tolerance to dietary sucrose and, in two cases, mucosal biopsy results were available which demonstrated diminished sucrase activity by Dahlqvist-assay. The substudy was performed by comparison of ^13^CO_2_ appearance in the breath with the timed appearance of ^13^C-monosaccharides in the plasma, the primary products of sucrose digestion.

Successful sucrose digestion results in absorption of ^13^C-fructose and ^13^C-glucose, for which ^13^C-fructose is mostly taken up by the liver on first-pass portal circulation while most resultant ^13^C-glucose passes to the systemic circulation for immediate use by vital organs [34]. Blood sugar samples were then obtained, and comparisons were made to indirectly assess sucrose digestion independent of mucosal biopsies. Concurrently, plasma samples were obtained with timed biphasic ^13^C-sucrose breath test sampling at 30, 60, and 75 minutes after substrate ingestion and assayed. The breath samples were analyzed for ^13^CO_2_ enrichment by infrared mass dispersive spectrophotometry. The appearance of ^13^C-plasma glucose was determined by yeast fermentation of plasma and expression of ^13^CO_2_ with comparison against a reference substrate standard fermentation [35, 36]. Resultant gas samples were quantitatively eluted from reaction vessels and similarly analyzed for ^13^CO_2_ enrichment, and both outcomes were compared.

Subject-A was a healthy, asymptomatic 45.9-year-old woman that routinely ingested dietary sucrose without adverse reactions; her SI genotype (WT/WT) revealed no genetic aberrations (ABI PRISM SNaPshot Assay Life Technologies, performed by LabCorp test 511570 SI, RTP, North Carolina). Subject-D was a profoundly symptomatic 46.7-year-old woman that avoided dietary sucrose and required sacrosidase supplementation with most meals to avoid typical symptoms of maldigestion; her SI genotype revealed homozygous aberrations [c.3218G>A (p.G1073D)]. A recent mucosal biopsy revealed no sucrase activity (only one sample obtained). Subject-B was a moderately symptomatic 23.3-year-old woman that restricted dietary sucrose to avoid typical symptoms. She was the biologic daughter of Subject-D and her sucrase-isomaltase genotype revealed heterozygous aberrations [c.3218G>A; (p.G1073D)/WT]. She has not required sacrosidase supplementation and she manages her symptoms by dietary sucrose restriction. Subject-C was a moderately symptomatic 43.9-year-old woman with a different SI mutation [c.5234T>G; (p.P1745C)] that also restricted dietary sucrose to avoid typical symptoms and periodically required sacrosidase supplementation. A sucrase assay of mucosal biopsy performed 13 years earlier demonstrated diminished sucrase activity (25 *μ*m/min/gm protein: exactly at the diagnostic threshold, 5th percentile).

The plasma samples were processed in a closed reaction system where aliquots of plasma (1.00 mL) were incubated (37°C) overnight with a 20 mL culture of baker's yeast (Red Star, Cedar Rapids, IA). Each test tube was fitted to a standard 250 mL Mylar breath collection bag and each system was primed with 20.0 mL oxygen (100%). After 24 hours of elapsed time, the system was purged with 100.0 mL of carrier gas containing 3% unlabeled (^12^C) carbon dioxide. Samples were analyzed for ^13^CO_2_ enrichment by comparison with a reference standard using mass dispersive spectrophotometry, adjusted for individual plasma volume (DOB *∗* PV), and results were stratified according to genotype.

### 2.7. Statistical Analysis

Deidentified clinical data were abstracted from the Baylor Clinic EPIC health care software (*Epic Systems*; Verona, Wisconsin). Clinical and experiment data were collected and transcribed to spreadsheets (*Excel*,* Microsoft*, Redmond, WA). Statistical analyses utilized an online statistical application [*VassarStats* (http://vassarstats.net/)]. Graphs and figures were prepared using DeltaGraph 6 (*Red Rock Software, Inc.*, Salt Lake City, UT).

### 2.8. Generation of Diagnostic Cut-Off Value

We were unable to directly validate the adult control group using comparative enzyme assays on biopsy sample; therefore, a diagnostic cut-off point for the breath test was determined by calculating the lower 95% confidence interval of ^13^CO_2_ enrichment data obtained for the asymptomatic control group (CGO-S cut-off ~ 85%). For the purpose of estimating a reference diagnostic cut-off point from normal subjects, a* single sample t-test* was used to determine the difference between the observed mean of the asymptomatic, historically sucrose-tolerant control sample, and the theoretically normal value (CGO-S = 1.00). The CGO-S values of the control group at 60-minute, 75-minute, and mean 60–75-minute time points were tested against the hypothetical CGO mean (60′: *p* = 0.09; 75′: *p* = 0.41; 60–75′: *p* = 0.15), and the data point to 75′ time point was the most telling. The lower limit of the 95% confidence interval (CGO-S) was determined for each time point.

## 3. Results

**Figure 1:**
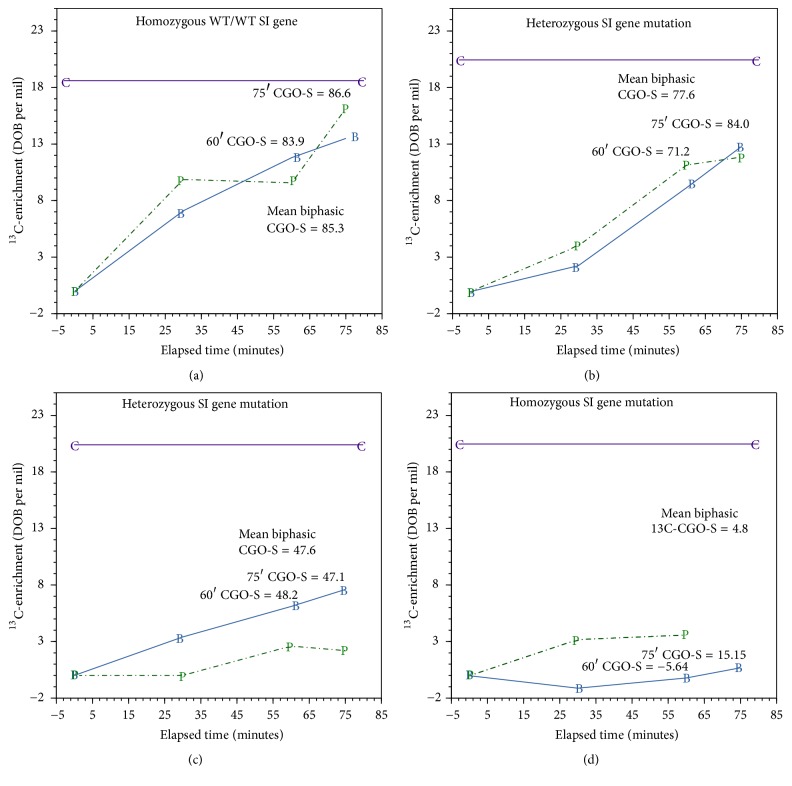
The four genetically SI-characterized (chromosome 3-NC_000003.12) subjects undertook ^13^C-S/GBT with simultaneous breath and blood sampling for plasma fermentation to release ^13^CO_2_ to validate breath sample enrichment outcomes; Phase I ^13^C-sucrose oxidation data and plasma fermentation data are shown. (a) 45.9 y/o healthy, asymptomatic woman without suspected SI gene mutation and normal CGO for ^13^C-sucrose (>80%); (Chr. 3q25.2-26.2) WT/WT. (b) 23.3 y/o symptomatic woman with heterozygous SI gene mutation and abnormal mean CGO for ^13^C-sucrose (<80%); Chr. 3q25.2-26.2 [c.3218G>A (p.G1073D)/WT]. (c) 53.9 y/o symptomatic woman with heterozygous SI gene mutation and abnormal mean CGO for ^13^C-sucrose (<80%); Chr. 3q25.2-26.2 [c.5234T>G (p.P1745C)/WT]. (d) 46.7-year-old symptomatic woman with homozygous SI gene mutation and abnormal (very low) mean CGO for ^13^C-sucrose (c/o < 80%); Chr. 3q25.2-26.2 [c.3218G>A (p.G1073D)/c.3218G>A (p.G1073D)]. B: ^13^CO_2_ breath sample enrichment (delta per mil); P: plasma sample ^13^CO_2_ enrichment (delta per mil); C: theoretical target ^13^CO_2_ plasma enrichment derived from* in vitro* reference substrate (^13^C-glucose 3 mg/L).

The four genetically SI-characterized subjects undertook ^13^C-S/GBT with simultaneous breath and blood sampling to validate breath test outcomes. High enrichment values, derived from effective sucrase activity, resulted in the appearance of high ^13^CO_2_ tracer from the plasma and vice versa. Results are shown in Figure 1. Panel (a) (Subject-A: genotype WT/WT) breath enrichment was compared with blood test enrichment. The data closely paralleled each other and were markedly higher than the results obtained from Subject-D (SI homozygous mutation genotype c.3218G>A), whose results for the sucrase portion of the test approached zero. As predicted, the results for the heterozygous subjects (B & C) were depressed as compared to Subject-A and were markedly improved over the data obtained for the SI-affected Subject-D. The intermediate results (B & C) were consistent with those to be expected for the heterozygous subjects.

**Figure 2:**
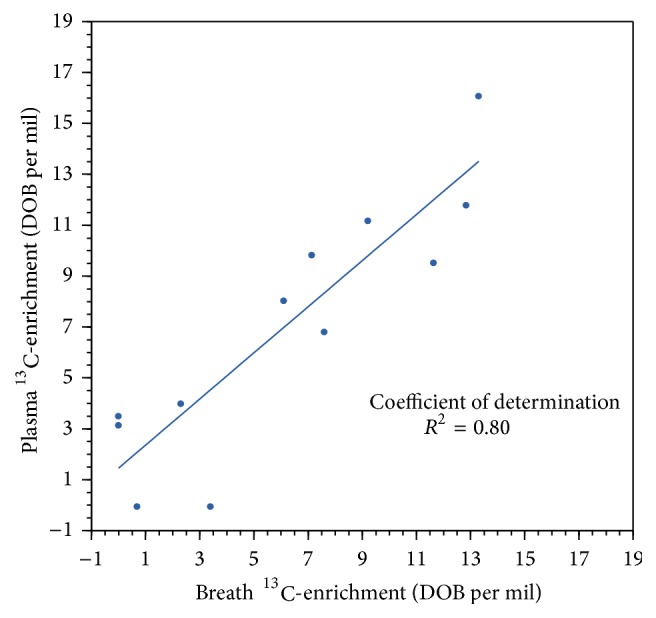
To validate breath sample enrichment outcomes, the relationship between ^13^CO_2_-breath sample oxidation data and ^13^CO_2_-plasma fermentation data was determined for the four genetically SI-characterized subjects (chromosome 3-NC_000003.12). Subjects undertook ^13^C-S/GBT with simultaneous blood sampling for plasma glucose fermentation to release ^13^CO_2_. Phase I ^13^C-sucrose oxidation data and plasma fermentation data are shown.

Collectively, the results obtained from the blood assay (fermentation products of digestion) closely correlated (*r* ^2^ = 0.80) with ^13^CO_2_ breath enrichment (Figure 2).

**Figure 3:**
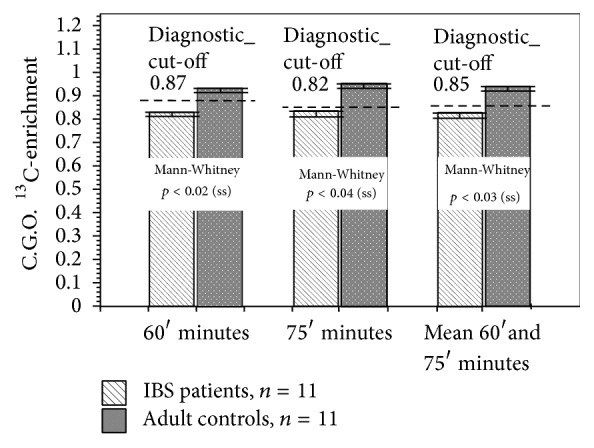
Between-group comparisons of biphasic ^13^C-sucrose/^13^C-glucose breath test data obtained from symptomatic patients with FBD and asymptomatic adult control subjects. Patients with FBD demonstrated decreased coefficient of glucose oxidation values for ^13^C-sucrose at 60-, 75-, and mean 60–75-minute time points when compared with asymptomatic controls.

Eleven patients with FBD and 11 paired control subjects were enrolled and underwent the ^13^C-S/GBT (Figure 3). The primary data sets, on which the conclusions presented herein rely, are appended (see Supplemental Table 1 in Supplementary Material available online at http://dx.doi.org/10.1155/2016/7952891). There were no adverse reactions observed or reported during the study. Seven patients (64%) showed decreased sucrose digestion with CGO-S cut-off values below 87% at the 60-minute time point (mean CGO-S = 0.80; 95% CI = 64–96%) and seven FBD patients (73%) showed decreased sucrose digestion with values below 85% at the 75-minute time point (mean CGO-S = 0.82; 95% CI = 62–98%) and eight patients showed decreased sucrose digestion with values below 85% at the mean 60–75-minute time point (mean CGO-S = 0.85; 95% CI = 85–100%).

Three subjects experienced typical, mild abdominal bloating and abdominal discomfort during the test. In sharp contrast, only one control subject (9%) demonstrated diminished sucrose digestive capacity at the 60-minute, 75-minute, and 60–75-minute time points but did not report any adverse symptoms. Using the derived breath test diagnostic CGO-S cut-off value of 0.85 at the 60–75-minute time points, a Fisher Exact Probability Test of proportions discriminated between the groups (*Chi Statistic: 6.55; p* < 0.02).

^13^CO_2_ Enrichment Coefficient of Glucose Oxidation for ^13^C-Sucrose (CGO-S) data values obtained at 60-minute, 75-minute, and the mean 60–75-minute values are shown in Supplemental Table 1.

## 4. Discussion

Free sucrose is a relatively modern component to the human diet. Sucrose is pervasive in commercially prepared foods, is frequently added to drinks and recipes, and is, therefore, difficult to avoid in the habitual diet. The specific association between sucrose intolerance and FBD-like symptoms has been previously observed [37] but has not been well evaluated. Like lactose, the opportunity for dietary sucrose to be included among the causes of abdominal discomforts is present, and testing is a key component to the therapeutic approach. This small pilot study indicates that sucrose maldigestion could be a factor in some cases of FBD, especially with more difficult cases. This finding was exemplified by eight of the eleven patients with FBD who demonstrated diminished sucrase activity by breath testing. Due to cost constraints, the affected patients could not be assessed by genotyping or mucosal biopsy assays. However, the parallel blood testing for ^13^C-labeled products of sucrose digestion in the four patients with SI gene mutations, used as a surrogate for assay of random intestinal biopsies, successfully validated use of ^13^C-S/GBT.

Approximately two-thirds of the glucose and one-quarter of the fructose (or half of the total stable isotopic-tracer load) were expected to appear in the systemic circulation and to be available as a proportional indicator of normal digestion since very little free sucrose is passively absorbed by the intestines. After fasting, most absorbed fructose is converted to trioses in the liver over time, and trioses are subsequently released into the systemic circulation as lactate for oxidation in extrahepatic tissues or stored as glycogen. Plasma content of ^13^C-labeled monosaccharides closely paralleled breath sample enrichment as the appearance of ^13^CO_2_ in either breath or plasma and are both dependent upon mucosal sucrase activity. As such, ^13^CO_2_ breath sample enrichment is a valid measure of sucrase activity.

We note that reference values for mucosal disaccharidase assay activities are also determined by mathematical analysis of the distribution of the means of thousands of samples (*all comers*) assayed over a long period and were not by stratified comparison with symptoms [38]. Furthermore, random biopsies of proximal duodenum may not be representative of true digestive capacity, which occurs distally just beyond the Ligament of Treitz. Therefore, validity would be difficult to ascribe using an endoscopic approach. Sucrose maldigestion may be qualitatively tested by dietary challenge-load testing and assay or breath testing for bacterial-derived, expired methane and hydrogen [39, 40]. Elimination diets and food records are tedious, but they appear to be somewhat helpful in the evaluation of sucrose maldigestion [41]. At the time of this writing, it is difficult to precisely correlate symptoms with CGO-S values in patients since the cohort sample size was small.

A monophasic ^13^C-sucrose breath test was previously applied to a rat model with chemotherapy-induced gastrointestinal mucositis and varying degrees of sucrose activity [42], but that test is not directly applicable for human use because it did not correct for individual differences in metabolic activity and the carbon dioxide production rate. A biphasic ^13^C-sucrose/glucose breath test (^13^C-S/GBT) was successfully used to stratify homozygous, heterozygous, and compound heterozygous cases of congenital sucrase-isomaltase insufficiency [43]. That test had been validated in children and adolescents by comparison with sucrase assay of endoscopic mucosal biopsies. It was also observed that cachectic patients, with cancer and FBD-like symptoms, have markedly diminished ^13^C-S/GBT oxidation scores that worsen with chemotherapy (unpublished). As such, the new test reported on herein has greater utility, particularly since improvements have been made beyond the currently marketed test [44]. In a way similar to the breath-hydrogen test, it is unlikely that the biphasic ^13^C-S/GBT could be fully validated since gastroenterologists that treat adults are reluctant to perform small bowel biopsies for disaccharidase activity. Nevertheless, the implication of this study is large and points to the need for a stronger study with comparative ^13^C-blood-sugar assays using gas chromatographic mass spectrometry, which might be superior to invasive approaches.

The findings of this study also point to the need for clinicians to carefully obtain a dietary history during the evaluation, admittedly a time-consuming task that in this day and age could be initially approached using a handheld device application if a dietician is not readily available [45]. Rao et al. noted that in recent years sucrose consumption had significantly increased and that the source of sweetener had changed from cane sugar (i.e., sucrose, a disaccharide of glucose, and fructose) to corn sweetener (primarily equimolar quantities of sucrose and fructose) [46]. Levels of fructose consumption have increased by more than 1000% between 1970 and 1990 and this suggests that a relatively limited distal absorptive capacity and overindulgence might be a factor in the FBD constellation [47]. Rao et al. also observed a distinct improvement in FBD symptoms when the fructose dose was decreased to less than 25 grams [46]. This finding suggests that recognition of dietary fructose intolerance might be clinically useful, but it remains unclear if digestion, absorption, or both mechanisms could be at fault. Of note, there has been one recent report of symptomatic relief with the use of empiric pancreatic enzymes, which proposes to potentiate residual, downstream mucosal disaccharidase activity and might point to the need for further diagnostic testing [42].

As predicted, the sucrose load used in this study was well tolerated but does not eliminate the possibility of fructose intolerance that could be confirmed by additional ^13^C-fructose testing [48]. An abnormal ^13^C-S/GBT should also prompt evaluation for fructose malabsorption. The sucrose load might be increased for subsequent studies, but responding to this criticism is at the risk of delaying gastric emptying by increasing caloric load.

The concern over excessive amounts of maldigested sucrose contributing to the FBD condition has been the subject of some debate [49]. The role of sucrase insufficiency has not been adequately tested in adult FBD patients whose gastrointestinal evaluations have seldom included direct-biopsy disaccharidase assays. Clinicians might consider dietary sucrose restriction and assess symptom response as a means of evaluation and, if sucrase insufficiency is suspected, obtain a consultation with a dietician to help minimize sucrose exposure. Our finding of sucrase insufficiency in FBD by ^13^C-sucrose breath testing was not surprising since the patients that were recruited for participation had resistant symptoms, and we had observed these epiphenomena before in children. However, our expectation was that the diagnostic yield would be lower. Our group's earlier pediatric experience involved the use endoscopic biopsy assays and demonstrated that sucrase deficiency occurs in 28% of children presenting with unexplained subacute or chronic abdominal pain that encompassed FBD-like symptoms [50]. This study's outcome is higher than the frequency of known sucrase mutations, and our findings suggest that the sucrose intolerance may be acquired or that epigenetic factors may be in play. This present study was not designed to investigate the cause of sucrase insufficiency, which could be genetic [51, 52], acquired due to inflammation, cachexia, and anticancer therapy [53] or specifically related to the fructose component or the GLUT-5 transporter that might be involved [54].

Since the 1980s, various sucrose-load breath tests have been used as a means to estimate intestinal absorptive function and sucrase activity in humans, rodents, and swine [55], and this approach falls into the realm of metabolomics, a field that is still expanding. This study demonstrated the feasibility to apply the test clinically. Earlier tests assessed changes in carbon isotopic enrichment as a metabolic indicator but were of limited value because they solely relied upon naturally enriched substrates and the use of cumbersome mass spectrometry [56, 57]. Further refinements to ^13^C-breath test technologies incorporate highly enriched stable isotope tracers and are based on the principle that intake of ^13^C-labeled substrates is expected to be predictably metabolized to ^13^CO_2_ and that the measured increase of ^13^CO_2_ above baseline proportionally reflects the targeted metabolic functions [58–60]. The use of stable isotope tracers with mass dispersion spectrophotometry also provides convenient point-of-care functionality to the breath test in a typical outpatient scenario, an advancement that has made ^13^C-breath testing affordable and convenient at the bedside [61]. Furthermore, infrared spectrophotometric breath testing can be performed safely in an outpatient center with minimal personnel training necessary to conduct analyses [62].

The modus of the biphasic ^13^C-S/GBT exploits the principle that the constituents of sucrose (glucose and fructose) should be promptly oxidized after ingestion and undergo glycosidic hydrolysis, portal absorption, and systemic oxidation such that the timed appearance and magnitude of ^13^CO_2_ recovery from breath sampling provide a good assessment of the digestive process. Modified Scofield equations have been used in the past to adjust ^13^C-urea breath test outcomes in children [63], but that application appears to best relate to bacterial urease in the stomach lumen. However, significant technical differences exist such that onerous mathematical models would be needed to apply that approach here [64]. In this study, the digested components of sucrose were oxidized and the resultant, exhaled carbon dioxide (^13^CO_2_) was used as a quantifiable correlate of hydrolysis. However, an innovative adjustment approach, using superdosed ^13^C-glucose, was also used to correct for individual variations in habitus known to affect metabolic rate. (e.g., individuals, with normal digestive activity and increased body mass index would be expected to yield proportionally low tracer signals as compared to lean individuals). This advancement was implemented because ^13^CO_2_ enrichment derived from sucrose directly provides no information on retained tracer in body enrichment pools and fluxes [65]. Considering that normal substrate-oxidation peaks early and begins to diminish slowly in concentration, the relatively stable isotopic flux condition, after 75 minutes, provided the opportunity to piggyback a secondary, superdosed ^13^C-glucose breath test, using the same stable isotope tracer; proportional adjustments were then made. The use of ^13^C-glucose provided for a direct and practical adjustment approach that was not dependent on mucosal disaccharidase, and when paired with oxidation results of the target ^13^C-sucrose substrate, individualized mucosal enzyme activity was measured.

The application of a secondary, superdosed ^13^C-glucose breath test also addressed the issue of variable habitus, leaving only mucosal sucrase activity as the isolated physiological difference. In theory, ^13^C-glucose undergoes the same metabolic fate as the hydrolyzed sucrose-derived glucose and fructose monomers but requires no further digestion before oxidation [66, 67]. Therefore, oxidation results were expressed as a proportional change in ^13^CO_2_ enrichment ratio between the target ^13^C-sucrose substrate and the change in ^13^CO_2_ enrichment associated with the ^13^C-glucose substrate and reported as the Coefficient of Glucose Oxidation for Sucrose (CGO-S) with 100% (1.00) being theoretically ideal. Since the second phase test was applied under the same conditions, it effectively removed the need to consider confounders, such as intercurrent illness, activity changes, fasting status, and metabolic flux, because such effects are conceptually negated (*cancelled out*). In the sucrase-insufficient condition, delayed digestion would result in a diminished primary sucrose-related oxidation value and isotopic-carryover into the subsequent ^13^C-glucose test period and increase the divisor value. This is a subtle, but important consideration, since initial ^13^CO_2_ recovery is attributed to proximal small bowel sucrose digestion and oxidation.

Delayed digestion is expected to contribute to distal fermentation and symptoms related to the production of small molecule effectors, such as N-acyl-3-hydroxypalmitoyl-glycine (commendamide) [68]. In the cases of sucrase insufficiency and delayed digestion, aberrant enrichment carryover would be expected to accentuate the resultant glucose oxidation data (divisor) and theoretically increase the sensitivity of the test and possibly decrease the test specificity. However, the carryover effect is substantially diminished after mathematical adjustment for the superdosing (x/5). As such, any significant ratio discrepancy from unity could be attributed to faulty sucrose digestion at the mucosal level in the absence of other pathologies, such as gastroparesis.

The diagnostic cut-off value used in this study (<0.85) was derived indirectly by determining 95% confidence intervals bracketing the breath test outcomes of the symptom-free control group. However, this diagnostic cut-off point should be considered tenuous at this time, and the issue should be revisited by a larger, direct comparative study of nonmutants with endoscopic mucosal sucrase assays and/or blood enrichment assays.

## 5. Conclusions

Sucrase deficiency appears to be a factor in the FBD condition. ^13^C-S/GBT was successfully used to assess sucrose digestion and the results reported herein support the need for a larger study. Sucrose maldigestion may also be an indicator of a larger issue, starch maldigestion. This is considered since isomaltase is concurrently expressed with sucrase (same gene product 3q25.2-q26.2) and because starch is the major dietary staple that is difficult to limit. A careful history identifying a link between GI symptoms and starchy food intake, in addition to sucrose intake, should be helpful in predicting the contribution of dietary intolerances to recurrent GI symptomatology [69]. Like sucrose, for adults suffering from carbohydrate malabsorption, a low fermentable diet might be considered in therapeutic planning since maldigestion may promote colonic proliferation of saccharolytic taxa [70]. It raises the possibility that sacrosidase [43], amyloglucosidase [71, 72], or other enzyme supplementation [73] might benefit some patients with FBD or cachexia-induced maldigestion [74], for which further studies are also needed.

## Supplementary Material

The primary data are tabulated in Supplemental Table 1 and stratified according to clinical condition. Data include coded identifiers, age, BMI, 60 minute CGO-S values, 75 minute CGO-S values, and mean 60-75 minute CGO-S values. Descriptive statistics are provided.

## Figures and Tables

**Table 1:** Summary of the clinical features of 11 subjects diagnosed with functional bowel disorder as per gastroenterologist (HTS). All patient subjects experienced recurrent feeling of bloating or visible distension for at least 3 days/month during the prior 3 months and did not meet criteria for a diagnosis of functional dyspepsia, irritable bowel syndrome, or other functional GI disorders. All symptomatic subjects were scored as “severely affected” in accordance with the 2011 Rome Foundation working-team report [14]. None of the asymptomatic control subjects met any criteria for functional bowel syndrome of any type (*data not shown*).

Number	Gender	BMI	75′ breath test CGO	Predominant symptoms at least 3 days per month, >6 months [14]	Pertinent history	Stool pattern	Symptoms during or immediately after breath test
1	F	19	83.4	Abdominal pain, bloating, increased flatus; severity: severe	Lactose intolerant (avoids all lactose), poor response to FODMAP diet & VSL number 3	Soft, irregular frequency, difficult to evacuate, no constipation, no diarrhea	None reported beyond baseline FBD symptoms

2	F	25	57.0	Abdominal pain, bloating, increased flatus; severity: severe	Good response to FODMAP diet	Irregular frequency, difficult to evacuate, no constipation, no diarrhea	None reported beyond baseline FBD symptoms

3	M	28	83.8	Abdominal pain, bloating, increased flatus; severity: severe	Some pain relief with eating, but not predictable	Irregular frequency, fluctuating consistency, no constipation, no diarrhea	Increased A/P and bloating near test completion

4	F	42	75.1	Abdominal pain, bloating, nausea, increased flatus;severity: severe	Marginal response to FODMAP diet	Irregular frequency, fluctuating consistency, no constipation, no diarrhea	Increased A/P and bloating near test completion

5	F	31	72.5	Abdominal pain, bloating; severity: severe	Benign other history	Irregular frequency, fluctuating consistency, no constipation, no diarrhea	None reported beyond baseline FBD symptoms

6	M	35	78.3	Abdominal pain, bloating; severity: severe	History of diverticulitis	Irregular frequency, fluctuating consistency, no diarrhea	None reported beyond baseline FBD symptoms

7	F	26	58.0	Abdominal pain, bloating; severity: severe	Benign other history	Irregular frequency, fluctuating consistency, no diarrhea	None reported beyond baseline FBD symptoms

8	F	33	138.8	Abdominal pain, bloating, increased flatus; severity: severe	Lactose intolerant (avoids all lactose)	Irregular frequency, fluctuating consistency, no constipation, no diarrhea	None beyond baseline FBD symptoms

9	F	25	77.3	Abdominal pain, bloating; severity: severe	History of rectal carcinoid, resolved	Irregular frequency, fluctuating consistency, no constipation, no diarrhea	Increased A/P and bloating near test completion

10	F	25	75.4	Abdominal pain, bloating; severity: severe	Benign other history	Irregular frequency, no constipation, no diarrhea	None beyond baseline FBD symptoms

11	M	20	45.6	Abdominal pain, bloating, increased flatus (significant); severity: severe	Good response to low carbohydrate diet, strong family history of similar issues	Increased frequency, frequent loose stool (not frank diarrhea), no constipation	None beyond baseline FBD symptoms

## Abbreviations

BT: Breath test
^13^C-S/GBT: ^13^Carbon-sucrose/^13^C-glucose breath test
CGO-S: Coefficient of Glucose Oxidation for Sucrose
DOB: Delta‰ over baseline
FBD: Functional bowel disorder
GI: Gastrointestinal
IBS: Irritable bowel syndrome
NSD: Not significantly different
PV: Plasma volume in liters
SI: Sucrase-isomaltase
WT: Wild type.
