# Supplementary material for: Use of the Biphasic 13C-Sucrose/Glucose Breath Test to Assess Sucrose Maldigestion in Adults with Functional Bowel Disorders
Source: Biomed Res Int. 2016 Aug 8;2016:7952891. doi: 10.1155/2016/7952891 (PMC4992795; doi:10.1155/2016/7952891)
Supplement: Supplementary file 1 — The primary data are tabulated in Supplemental Table 1 and stratified according to clinical condition. Data include coded identifiers, age, BMI, 60 minute CGO-S values, 75 minute CGO-S values, and mean 60-75 minute CGO-S values. Descriptive statistics are provided. [file 7952891.f1.pdf]

<u>Supplement Table 1 (Primary Data Set)</u>. The $^{13}CO_2$ Enrichment Coefficient of Glucose Oxidation for ^{13}C-Sucrose (CGO-S) values obtained at 60 minutes, 75 minutes and the mean 60-75 minute values are tabulated for all adult subjects diagnosed with functional bowel disorder (left) and asymptomatic controls (right), N=11 each. The CGO-S value is the ratio of enrichment (*delta over the baseline*) derived from digestion and oxidation of ^{13}C-Sucrose (25 mg.) and from the oxidation of ^{13}C-glucose (super dose 125mg) divided by 5. Diagnostic cut-off values for each time point were determined from the 95% confidence interval lower-limit.

Adults with Functional Bowel Disorder Subject #	Age	BMI	^{13}CO2 Enrichment Coefficient of Glucose Oxidation for ^{13}C-Sucrose at 60'	^{13}CO2 Enrichment Coefficient of Glucose Oxidation for ^{13}C-Sucrose at 75'	^{13}CO2 Enrichment Coefficient of Glucose Oxidation for ^{13}C-Sucrose at 60'-75' Mean
**FBD-1**	46.3	18.88	**0.77**	0.83	**0.80**
**FBD-2**	29.4	25.06	**0.57**	**0.57**	**0.57**
**FBD-3**	29.6	28.17	0.87	0.84	0.86
**FBD-4**	51.6	41.81	1.32	1.35	1.33
**FBD-5**	24	31.32	**0.79**	**0.72**	**0.76**
**FBD-6**	47.1	35.43	**0.58**	**0.78**	**0.68**
**FBD-7**	66	25.79	**0.55**	**0.58**	**0.57**
**FBD-8**	60.3	32.54	1.13	1.39	1.26
**FBD-9**	33.9	24.45	0.92	**0.77**	**0.84**
**FBD-10**	28.7	24.56	**0.83**	**0.75**	**0.79**
**FBD-11**	49.8	20.34	**0.47**	**0.46**	**0.46**
**Mean**	42.43	28.03	0.80	0.82	0.81
**STD Dev**	14.09	6.76	0.26	0.29	0.27
**STD_ERR**			0.08	0.09	0.08
**95%CI**			0.80±0.16+	0.82±0.18	0.81±0.16

Adult Control Subject #	Age	BMI	^{13}CO2 Enrichment Coefficient of Glucose Oxidation for ^{13}C-Sucrose at 60'	^{13}CO2 Enrichment Coefficient of Glucose Oxidation for ^{13}C-Sucrose at 75'	^{13}CO2 Enrichment Coefficient of Glucose Oxidation for ^{13}C-Sucrose at 60'-75' Mean
**ACS-1**	60.8	29.83	1.00	0.92	0.96
**ACS-2**	53.2	21.45	1.10	0.94	1.02
**ACS-3**	22.9	19.15	0.90	0.82	0.86
**ACS-4**	57.4	27.13	**0.74**	**0.82**	**0.78**
**ACS-5**	22.8	21.56	1.47	1.24	1.36
**ACS-6**	22.8	18.56	0.89	0.75	0.82
**ACS-7**	24.6	18.74	0.91	0.96	0.94
**ACS-8**	32.2	21.18	1.07	0.77	0.92
**ACS-9**	25.6	22.27	0.92	0.93	0.93
**ACS-10**	53.3	25.05	0.94	0.89	0.91
**ACS-11**	50.6	23.79	1.03	0.90	0.97
**Mean**	38.75	22.61	1.00	0.90	0.95
**STD Dev**	16.03	3.58	0.19	0.13	0.15
**STD_ERR**			0.06	0.04	0.05
**95%CI**			1.00±0.12	0.90±0.08	0.95±0.10
**Lower Limit Cutoff**			**0.87**	**0.82**	**0.85**

7952891 Opekun
